# A clinical study of the effects of lead poisoning on the intelligence and neurobehavioral abilities of children

**DOI:** 10.1186/1742-4682-10-13

**Published:** 2013-02-18

**Authors:** Shuangxing Hou, Lianfang Yuan, Pengpeng Jin, Bojun Ding, Na Qin, Li Li, Xuedong Liu, Zhongliang Wu, Gang Zhao, Yanchun Deng

**Affiliations:** 1 Department of Neurology, Xijing Hospital, Fourth Military Medical University, No.15 West Changle Road, Xi’an, 710032, China; 2 Department of Neurology, The 2nd Affiliated Hospital of Xi’an Medical College, No.167 Fangdong Road, Xi’an, 710038, China; 3 Department of Physiology, Wenzhou Medical College, Wenzhou, 325000, China

**Keywords:** Child, Lead poisoning, Intelligence development, Neurobehavioral ability

## Abstract

**Background:**

Lead is a heavy metal and important environmental toxicant and nerve poison that can destruction many functions of the nervous system. Lead poisoning is a medical condition caused by increased levels of lead in the body. Lead interferes with a variety of body processes and is toxic to many organs and issues, including the central nervous system. It interferes with the development of the nervous system, and is therefore particularly toxic to children, causing potentially permanent neural and cognitive impairments. In this study, we investigated the relationship between lead poisoning and the intellectual and neurobehavioral capabilities of children.

**Methods:**

The background characteristics of the research subjects were collected by questionnaire survey. Blood lead levels were detected by differential potentiometric stripping analysis (DPSA). Intelligence was assessed using the Gesell Developmental Scale. The Achenbach Child Behavior Checklist (CBCL) was used to evaluate each child’s behavior.

**Results:**

Blood lead levels were significantly negatively correlated with the developmental quotients of adaptive behavior, gross motor performance, fine motor performance, language development, and individual social behavior (*P* < 0.01). Compared with healthy children, more children with lead poisoning had abnormal behaviors, especially social withdrawal, depression, and atypical body movements, aggressions and destruction.

**Conclusion:**

Lead poisoning has adverse effects on the behavior and mental development of 2–4-year-old children, prescribing positive and effective precautionary measures.

## Background

Lead exists widely in the environment, and it is a heavy metal element with neurotoxic effects. In contrast to trace elements such as iron and zinc, lead has no know beneficial effects in the human body [1]. With the economic development of China in recent years, the concentration of lead in the environment has increased, and lead pollution has become a serious problem. Chinese people have begun to place greater scrutiny on the influence of lead exposure on human health, especially its impact on teenagers [2]. Lead poisoning can damage and produce irreversible harm to fetal growth, the growth of teenagers, and psychological, behavioral, and cognitive development [3,4].

Because lead in the environment gradually accumulates in the body, lead is regarded as one of the most harmful environmental toxins to toddlers [5]. When the blood lead level reaches about 50 μg/L in the body of children, it can impair growth, memory, intelligence, and behavior, even when there is no obvious clinical manifestation. The most important untoward effect of lead exposure is the impairment of the intelligence of infants and the learning abilities of children. Most recent investigations have verified that [6-8] lead exposure can affect learning and memory, and the intelligence quotient of children is inversely proportional to their blood lead level. Because childhood lead poisoning is quite common in China at present, it should be given adequate attention.

Most international long-term follow-up investigations of the effects of lead exposure on neurological dysfunctions in children have reported that these effects of lead are persistent, especially lead exposure in 2-year-old children, an age that appears to be a critical period for a child’s later intelligence quotient and academic achievement [9]. There is very strong evidence clearly indicating that lead has a negative influence on children's intelligence and behavioral development. The subjects in this study were mainly Chinese children between 2 and 6 years of age. Because of the age of the subjects, the universal intelligence development diagnostic scale of China (Gesell Development Schedules) [10] was used to assess intelligence development, and the most commonly used Achenbach Child Behavior Checklist (CBCL) [11,12] was used for the behavioral evaluations. In this study, we investigated the relationships between lead poisoning and intelligence and neurobehavioral changes in 2–4-year-old children, to provide a basis for clinical prevention and treatment measures.

## Subjects and methods

### Subjects

Ten children were randomly selected from each kindergarten in Xi’an. A total of 100 children were selected from ten kindergartens with an average age 2.8 years (SD = 1.45) and an age range of 2.2–3.9 years old. The exclusion criteria we used were (1) birthing problems such as delivery injuries or a low birth weight, (2) neonatal problems such as asphyxia, intracranial hemorrhage, hypoxic-ischemic encephalopathy, and severe jaundice, (3) acquired disabilities including poisoning, cerebral trauma, encephalitis, meningitis, encephalopathy and cerebral injuries following convulsions, and (4) potential factors influencing cognitive development such as malnutrition and inherited metabolic diseases. Based on these criteria, 96 children were selected to receive blood lead level detection tests.

### Determination of blood levels of lead

Blood levels of lead were determined by differential potentiometric stripping analysis (DPSA). A pre-coated mercury film method was used. Mercury film was pre-plated on the working electrode three times before detection, accumulating to a thickness that enabled it to work for 4 consecutive hours. Pb^2+^ and Hg^2+^ were enriched in the mercury film in selected conditions. Amalgam was stripped in a static mode. The content of Pb^2+^ and Hg^2+^ was quantified according to the peak of the stripping curve. The samples were detected without digestion.

Blood samples of 40 μL were collected and placed into 4 mL of de-ionized water. The color of the sample changed from purplish red to yellow after addition of 1.0–1.2 mol/L of a hydrochloric acid solution and 2.0 mL of a 0.01 mol/L KMnO_4_ solution. The color faded after addition of 0.1 mL of 50 g/L ascorbic acid. After 20 min, 75 μL × 10^-2^ mol/L of a mercury solution was added to the sample in a three-electrode system, and the peak was recorded. A blank reagent was processed at the same time. The lead content in the blood sample and reagent blank was calculated by a standard addition method, and the absolute difference between the two samples was regarded as the lead content.

The diagnostic criterion for lead poisoning in children was updated by the Centers for Disease Control and Prevention (CDC) in 2012 to a blood lead value of ≥50 μg/L, whether or not there are corresponding clinical signs or other blood biochemical changes. The children in this study were divided into a control group (blood lead value < 50 μg/L) and lead poisoning group (blood lead value ≥ 50 μg/L) according to this criterion.

### Questionnaire and behavioral assessments

The parents of the children in both groups were required to fill out a health questionnaire and CBCL for the children. The significance of this investigation and the project requirements were explained to the parents before they filled out the questionnaire, which was collected immediately. The content of the health questionnaire included approximately 40 questions related to source of exposure to lead, such as the family and social environment, life and health habit, and health conditions of the children. The main risk factors for high blood lead values could be assessed through questionnaire assignment analysis. A number of well-known instruments exist for assessing and categorizing the problem behavior of children. One of the most respected instruments in this area is the Child Behavior Checklist (CBCL; Achenbach, 1991). The CBCL is a paper-based questionnaire that presents caregivers with a series of 113 statements that relate to emotional and behavioral problems and competencies, using a 3-point response format to establish the frequency of problem behavior. Follow-up studies by the author of the CBCL (Achenbach, 1995; Achenbach & Howell, 1993) [13,14] and hundreds of studies relying on this instrument have reported adequate reliability and validity of CBCL scores in various populations, although there have been some challenges to this instrument (e.g., Raadal, Milgrom, Cauce, & Mancl, 1994) [15]. This study used the child and teenager psychological test software 2.0 to input and analyze the CBCL scores, and to provide measures of social withdrawal, depression, sleep problems, atypical body actions, previous medical diagnoses, aggression, and destruction.

### Intellectual development test

The development schedules used in this study were a set of four timetables devised by Arnold Gesell (1880–1961) at Yale University to evaluate the physical, emotional, and behavioral development of infants, toddlers, and preschoolers. They describe typical behavior at specific ages in the following areas: ability to adapt, motor functioning, use of language, and social interaction [16]. Here, the intellectual development test was conducted by professionals using the intelligence development diagnostic scale for children (0–6 years old) in subjects in both groups. A children’s intelligence development diagnostic scale called the Gesell Development Schedules as revised by the Beijing intelligence development group was used to assess the developmental quotients each child’s adaptive behavior, gross motor performance, fine motor movements, language development, and individual social behaviors.

### Statistical methods

All numerical variables in this experiment are expressed as means, standard deviations, minimum values, and maximum values. Enumeration data are expressed by case number, rate, and constituent ratio. Blood lead values between the experimental and control groups were compared using a *t*-test after logarithmic transformation (meet normal distribution). The cognitive development diagnostic scale results of the two groups were compared by *t*-test, the comparison of the prevalence of abnormal behavior in the two groups was conducted by a *χ*^2^ test, and scores on each behavior factor and the total behavioral score were analyzed by the rank-sum test. SPSS11.5 statistical software was used to analyze all data, and *P* < 0.05 was taken as being significant statistical difference. All test conducted were two-tailed, and the test statistic and its correspondent *P* value are shown.

## Results

### The relationship between blood lead levels and cognitive development in children

The blood lead value in 76 children was 4–246 μg/L, with the average blood lead value mean (±SD) of 88.39 (±67.92) μg/L. The log transformed blood lead values ranged 0.60–2.39, with an average blood lead logarithmic value mean (±SD) of 1.81 (±0.52). Scores on the child adaptive behavior developmental quotient, gross motor developmental quotient, fine motor movement developmental quotient, language developmental quotient, and individual- social behavior were 63–132, 69–135, 58–157, 59–138 and 68–136, respectively, with means (±SD) of 95.36 (±14.71), 96.03 (±14.68), 100.42 (±17.65), 92.08 (±16.95), and 107.54 (±14.15), respectively. Regression and correlation analysis (Table 1) showed that blood lead levels had an obvious negative correlation with the developmental quotients of child adaptive behavior, gross motor performance, fine motor movements, language development, and individual social behavior.

**Table 1:** Correlation analysis between blood lead levels and developmental quotients in children

	**Regression equation**	**F value**	***P*value**	**r value**
Adaptive behavior	y = 130.12–20.05x	7.23	<0.01	−0.845
Gross motor	y = 115.93–12.51x	6.95	<0.01	−0.553
Fine motor	y = 135.45–19.97x	7.01	<0.01	−0.709
Language	y = 130.11–21.47x	8.25	<0.01	−0.912
Individual social behavior	y = 129.13–16.26x	6.54	<0.05	−0.548

Regression equation: correspondent to blood lead logarithmic value.

r value: correspondent to blood lead logarithmic value.

Compared with the control group, scores on the measure of developmental quotients of child adaptive behavior, gross motor performance, fine motor movements, language development, and individual social behavior lagged in the children previously exposed to lead, at 20.15, 13.28, 17.82, 20.99, and 14.75, respectively. The comparisons (Table 2) between the two groups showed that there were significant differences on these 5 developmental quotients.

**Table 2:** **Comparison of childhood intelligence between the two groups** (**n** = **50**, mean ± SD)

**Measure index**	**Group**	**Score of measure index**	***t*value**	***P*value**
Adaptive behavior	Lead poisoning group	84.13 ± 9.87		
	Control group	104.28 ± 11.35	9.473	<0.01
Gross motor	Lead poisoning group	87.75 ± 12.02		
	Control group	101.03 ± 12.31	5.458	<0.01
Fine motor	Lead poisoning group	89.39 ± 13.78		
	Control group	107. 21 ± 16.44	5.874	<0.01
Language	Lead poisoning group	82.16 ± 12.87		
	Control group	103.15 ± 13.98	7.81	<0.01
Individual social Behavior	Lead poisoning group	93.41 ± 10.32		
	Control group	108.16 ± 12.24	6.515	<0.01

### The relationship between blood lead levels and behavior in children

The examination showed that there were 11 subjects with social withdrawal, 17 subjects with depression, 8 subjects with sleep problems, 6 subjects with atypical body actions, 34 subjects with aggressions, and 7 subjects with destructions among the 50 children in the lead poisoning group, and the rate of abnormal behavior was 27.7%. There were 7 subjects with social withdrawal, 4 subjects with melancholies, 5 subjects with sleep problems, 8 subjects with atypical body actions, 6 subjects with aggressions, and 5 subjects with destructions among the 50 children in the control group, and the rate of abnormal behavior was 11.7%. The rates of depression, aggressions, and abnormal behavior in the lead poisoning group were significantly higher than those in the control group (*P* < 0.01), and the rates of social withdrawal, sleep problems, aggressions, and destruction in the lead poisoning group were noticeably higher than those in the control group (*P* < 0.05). The prevalence of atypical body actions in the lead poisoning group was lower than that in control group (*P* > 0.05), and the prevalence of social withdrawal, sleep problems, atypical body actions, and destruction were significantly different between the two groups (*P* > 0.05) (Table 3).

**Table 3:** **Comparison of the prevalence of abnormal behaviors between the two groups** (**n** = **50**, %)

**Behavioral factors**	**Group**	**Normal behavior**	**Abnormal behavior**	***x***^**2**^**value**	***P*value**
Social withdrawal	Lead poisoning group	78.0 (39/50)	22.0 (11/50)		
	Control group	86.0 (43/50)	14.0 (7/50)	2.168	>0.05
Depression	Lead poisoning group	66.0 (33/50)	34.0 (17/50)		
	Control group	92.0 (46/50)	8.0 (4/50)	20.37	<0.01
Sleep problem	Lead poisoning group	84.0 (42/50)	16.0 (8/50)		
	Control group	90.0 (45/90)	10.0 (5/50)	1.591	>0.05
Body action	Lead poisoning group	88.0 (44/50)	12.0 (6/50)		
	Control group	84.0 (42/50)	16.0 (8/50)	0.664	>0.05
Aggression	Lead poisoning group	32.0 (16/50)	68.0 (34/50)		
	Control group	88.0 (44/50)	12.0 (6/50)	65.333	<0.01
Destruction	Lead poisoning group	86.0 (43/50)	14.0 (7/50)		
	Control group	90.0 (45/50)	10.0 (5/50)	0.758	>0.05

Comparison of behavior factor scores between the two groups (Table 4) showed that depression, atypical body actions, aggressions, and total behavior scores in the children in the lead poisoning group were significantly different than in the children in the control group (*P* < 0.01), and the most significant differences (*P* < 0.05) were in social withdrawal and destruction, without obvious differences in sleep problems between the two group (*P* > 0.05).

**Table 4:** **Comparison of behavior factor scores between the two groups** (**n** = **50**, **mean** ± **SD**)

**Behavior factors**	**Group**	**Behavior factor score**	**Z value**	***P*value**
Social withdrawal	Lead poisoning group	8.79 ± 5.41		<0.05
	Control group	6.42 ± 4.32	2.421	<0.01
Depression	Lead poisoning group	6.73 ± 4.67		
	Control group	3.76 ± 3.65	3.543	<0.01
Sleep problem	Lead poisoning group	3.87 ± 2.92		
	Control group	3.21 ± 2.68	1.177	>0.05
Body action	Lead poisoning group	5.72 ± 3.56		
	Control group	3.28 ± 2.59	3.919	<0.01
Aggression	Lead poisoning group	28.02 ± 11.69		
	Control group	20.01 ± 10.79	3.600	<0.01
Destruction	Lead poisoning group	7.43 ± 4.58		
	Control group	5.21 ± 3.91	2.607	<0.01
Total behavior score	Lead poisoning group	59.89 ± 26.74		
	Control group	41.66 ± 24.58	3.549	<0.01

### Risk factor analysis of lead poisoning in children

Among the more than 40 factors we examined in the questionnaire, the risk factors for lead poisoning in children included poor living habits such as finger-sucking, nail-biting, putting foreign objects in the mouth, playing with plasticine, and using colored tableware frequently. They also included eating puffed food, preserved eggs, and canned food, smoking by a family member, living near a large road, and recent room decoration. Furthermore, the educational background of the parents was negatively correlated with elevated blood lead levels in children, and the statistical analysis indicated OR > 1, *P* < 0.05. Dietary protein was a protective factor against lead poisoning (B < 0, OR = 0.276 < 1, *P* < 0.05). The results of the logistic regression analysis are summarized in Table 5.

**Table 5:** Logistic regression analysis of factors related to the prevalence of lead poisoning in children

**Independent variable**	**B**	**OR**	***P***
Well-educated parents	−0.81	1.75	0.049
Finger-sucking, nail-biting	0.556	1.743	0.041
Putting foreign objects in the mouth (e.g., pencil, toys)	0.44	1.26	0.029
Often eat puffed food, preserved eggs and canned food, etc.	1.623	4.837	0.008
Often playing plasticine, color pen, etc.	0.66	1.274	0.050
Using colored tableware frequently	0.87	2.011	0.038
Smoking by a family member	0.99	2.46	0.032
Recent room decoration	0.47	1.024	0.035
Living near main road	0.151	2.978	0.016
Daily protein intake	−1.42	0.276	0.033

## Discussion

Lead is a neurotoxin with no physiological functions in the human body, the ideal concentration of which in the blood is zero. However, because of the prevalence of lead in the environment, lead exists in the vast majority of human bodies. With the rapid development of industrialization, the production and use of lead, and its accumulative release, there has been a rapid increase in lead levels in the environment [17,18]. Children are very sensitive to lead poisoning because of their metabolism and growth characteristics. Lead can easily penetrate the brains of children because their blood–brain barriers are not fully developed. Research has shown that the blood–brain barrier of newborn rats is highly permeable to lead, and that the younger the subject is, the greater is the permeability. The amounts of lead penetrating the blood–brain barriers of 16-day-old and 26-day-old rats were 2.42- and 2.05-times, respectively, that of adult rats under the same conditions [19]. Lead poisoning can have continuous damaging effects on children, influencing the development of nervous, cardiovascular, digestive, urological, reproductive, and endocrine systems, with the most sensitive to lead poisoning being the nervous system [20,21].

That lead poisoning can impair the IQ of children has been verified by many Chinese and international scholars. Research by Schnass et al. [22] emphasized that the influence of blood lead levels in newborn children on a general cognitive index (GCI) gradually peaked at 1–3 years after birth, and this influence was most obvious on 4–5-year-olds. After studying 74 children at 4–14 years of age, Bellinger et al. [23] found that IQ is inversely proportional blood lead levels, and IQ values dropped about 6 points with every 100 μg/L increase in the level of lead in the blood. A high level of lead in the blood has adverse effects on intellectual development in children. A study by Miranda et al. implied that blood lead levels of 20–50 μg/L can impair the reading and math abilities of children [24]. Consistent with these results, we found that blood lead levels were significantly negatively correlated with the development quotients of children’s adaptive behavior, gross motor performance, fine motor performance, language development, and individual social behavior.

The earliest and most obvious influences of lead on the nervous system of children include neurobehavioral changes except for a decline in intelligence. It has been verified that lead exposure is associated with childhood behavior disorders [25]. Behavioral changes in 1–3-year-old children have been associated with low levels of lead exposure, which indicates that behavior disorders should be clinically monitored in children that have even low levels of lead exposure [26]. Lewendon et al. [27] found that blood levels of lead in children with behavior problems were higher than those in healthy children, and suggested that the blood lead content of children with behavior disorders should be monitored regularly. Mendola et al. [28] found low levels of perinatal lead exposure were associated with attention problems in children. Researchers also found recently that antisocial behavior, behavior disorders, and adolescent crime in childhood and adolescence are also related to lead exposure before and after birth [29-32]. The results of this study are consistent with those findings. There were significant differences between the scores for social withdrawal, depression, atypical body actions, aggressions, destructions, and total behavioral changes in children previously exposed to lead and the control group (*P* < 0.05), and there was no significant difference between these groups in sleeping problems. At present, most reports hold that blood lead levels have a relationship with behavior disorders in children, but no strong conclusions have been drawn yet as to which specific behaviors are influenced by lead exposure, which should be further studied [33,34]. Human intakes lead mainly from food, water and respiration. The human body intakes 100–300 μg of lead every day from the gastrointestinal tract, and in adults, about 10% of this is absorbed into the body, whereas the absorption rate of children can reach 40%. The half-life of lead in blood and rapid-exchange soft tissue is less than one month, whereas the half-life of lead phosphate in skeleton can be 20 years [35].

Based on these collective findings, as an environmental pollutant, lead can be regarded as a “risk indicator” for the evaluation of IQ development [36]. The exact relationship between lead exposure and IQ changes remains uncertain. However, IQ is negatively correlated with blood lead levels. Moreover, we found more behavior disorders in children exposed to lead than in normal children, especially in terms of social withdrawal, depression, atypical body actions, aggressions, and destruction. Therefore, future studies should use multivariate analysis to elucidate the influence of lead poisoning on childhood intelligence and behavior, as this may clarify the mechanisms underlying the untoward effects of blood lead, and more importantly draw attention to comprehensive screening of lead poisoning in children. High blood lead levels and lead poisoning can be prevented. Because lead dust in the environment is the main source of lead poisoning in children, children’s food and tableware should be covered by dust shields. Furthermore, we should ensure the dietary balance and supply of various nutrients for children, and help children develop good eating habits. Prevention, early detection, and early intervention of childhood blood lead poisoning can be achieved through environmental interventions, health education, screening, and focused monitoring.

## Consent

Written informed consent was obtained from the patient’s guardian/parent/next in keen for publication of this report and any accompanying images.

## Abbreviations

CBCL: Child Behavior Checklist; Cr2O3: Chromium oxide; CRM: Certified Reference Material; IQ: Intelligence quotient; PKC: Protein kinase C; MDA: Malondialdehyde.

## Competing interests

The authors declare that they have no competing interests.

## Authors' contributions

HSX and DYC carried out the studies and drafted the manuscript. YLF and LL designed the study and performed the statistical analysis. JPP and QN collected the questionnaires and conduct the analyses. DBJ and LXD conceived the study, participated in its design and coordination, and drafted the manuscript. WZL and ZG performed the experiments. All authors read and approved the final manuscript.
